# Emodin alleviates chronic constriction injury-induced neuropathic pain and inflammation via modulating PPAR-gamma pathway

**DOI:** 10.1371/journal.pone.0287517

**Published:** 2023-07-13

**Authors:** Ismail Badshah, Neelum Gul Qazi, Fawad Ali, Amber Mahmood Minhas, Arooj Mohsin Alvi, Mahmoud Kandeel, Muhammad Imran, Syed Shams ul Hassan, Simona Bungau

**Affiliations:** 1 Riphah Institute of Pharmaceutical Sciences, Riphah International University, Islamabad, Pakistan; 2 Department of Pharmacy, Kohat University of Science and Technology, Kohat, Pakistan; 3 Department of Biomedical sciences, College of Veterinary Medicine, King Faisal University, Al-hofuf, Al-Ahsa, Saudia Arabia; 4 Department of Biomedical Science, Pak-Austria Fachhochule: Institute of Applied Science and Technology, Mang Haripur, KPK, Pakistan; 5 Shanghai Key Laboratory for Molecular Engineering of Chiral Drugs, School of Pharmacy, Shanghai Jiao Tong University, Shanghai, China; 6 Department of Natural Product Chemistry, School of Pharmacy, Shanghai Jiao Ton University, Shanghai, China; 7 Department of Pharmacy, Faculty of Medicine and Pharmacy, University of Oradea, Oradea, Romania; University of New South Wales, AUSTRALIA

## Abstract

Neuropathic pain has been characterized as chronic pain resulting from pathological damage to the sensorimotor system. Because of its complex nature, it remains refractory to most of the therapeutic interventions, and surgical intervention and physiotherapy alongside steroidal treatments remain the only treatment protocols with limited success, hence solidifying the need to find efficacious therapeutic alternatives. Emodin was used as a post-treatment for its potential to be neuroprotective in the treatment of chronic constriction injury-induced NP. The first day following surgery, Emodin treatment began, and it lasted until the 21st day. On days 3, 7, 14 and 21, all behavioral investigations were conducted. The sciatic nerve and spinal cord were extracted for further molecular examination. Emodin elevated response latency, was able to delay the onset of mechanical hyperalgesia in rats on days 7, 14, and 21 and reduced the CCI-induced paw deformation. Emodin treatment significantly reduced lipid peroxidation and NO levels while restoring the GST, GSH and catalase. It significantly improved the disorientation of the sciatic nerve and spinal cord confirmed by H & E staining and reduced inflammatory markers as observed by the quantification of COX-2, TNF-α, p-NFκb and up-regulated PPAR-γ levels by ELISA and PCR. According to the findings, Emodin has antinociceptive and anti-hyperalgesic properties, which reduced pain perception and inflammation. We also suggested the involvement of PPAR-γ pathway in the therapeutic potential of emodin in chronic nerve injury.

## 1. Introduction

Neuropathic pain (NP) has been characterized as chronic pain resulting from pathological damage to the sensorimotor system [1]. It is of diverse and complicated nature depending upon the site of damage. Central lesions may result in spinal cord injury leading to paralysis or myelopathy; while peripheral lesions may lead to nerve injury featured by hyperalgesia or allodynia. It may arise as a result of excitability of primary sensory neurons in the peripheral nervous system causing exaggerated neuroplasticity [2]. Besides neuronal pathway, certain immune system components along with microglia and astrocytes induce exaggerated neuronal inflammation, contributing in the progression of NP [3]. Because of its complex nature, it remains refractory to most of the therapeutic interventions, and surgical intervention and physiotherapy alongside steroidal treatments remain the only treatment protocols with limited success, hence solidifying the need to find efficacious therapeutic alternatives.

Peroxisome proliferator-activated receptors are a member of ligand-gated transcription factors’ nuclear family which mediate actions of many G-protein coupled receptors, antioxidants and growth factors [4]. PPAR- γ specifically, plays various physiological roles as lipid metabolism and glucose homeostasis [5]. Mounting evidence suggests the involvement of PPAR-γ receptors in neuroprotection in ischemic injury and neurological diseases by promoting neuronal differentiation and neurite growth [6]. PPAR-γ also shows a striking therapeutic potential in treatment of chronic pain. It also shows promising anti-inflammatory potential in the brain by blocking amyloidogenic pathways [7]. PPAR-γ agonists as pioglitazone and rosiglitazone have been approved anti-diabetic agents [8] and have shown a potential to reduce inflammation by ameliorating TNF-α expression. Alleviation of thermal analgesia and mechanical allodynia by inhibiting astrocyte activation and activating Nrf2- pathway have been well documented [9, 10]. PPAR-γ antagonist as GW9662, abolish the analgesic and anti-inflammatory potential of PPAR-γ, eliciting pro-inflammatory response, giving an insight into the involvement of PPAR-γ mechanisms in NP [11].

Past few years have shown a steep inclination towards the use of natural compounds as therapeutic agents. Emodin is a natural anthraquinone derivative that has proven biological activities and pharmacological effects as antiallergic, antiviral, antidiabetic, antiulcer, antibacterial and immunosuppressant [12–15]. As a part of Chinese traditional medicine, it has been extensively used for the management and treatment of chronic pain and related co-morbidities. Various studies reported its neuroprotective effect in various neurodegenerative models as Alzheimer’s and ischemic stroke [16]. Emodin also alleviated NP induced by CCI model by mediating P2X(2/3) receptors in primary sensory neurons and modulating calcitonin-gene-related peptide in trigeminal neuralgia [17]. However, the molecular mechanisms underlying the effects of emodin on NP in spinal cord has been scarcely investigated. The current study, for the first time, suggests a potential involvement of PPAR-γ in the therapeutic potential of emodin in NP induced as a result of chronic constriction injury, in an attempt to present a probable pharmacological agent for the treatment and management of NP.

## 2. Chemicals and reagents

Emodin was procured from Carbosynth Limited, Berkshire, U.K. Suture 3/0 was acquired from Shifa International Hospital, Pakistan. Reagents as PBS tablets, proteinase K were purchased from MP-Biomedicals, USA. Tricholoroacetic acid (TCA), N-(1-Naphthyl) ethylenediamine dihydrochloride, Reduced glutathione (GSH), 1-chlor-2,4-dinitrobenzene (CDNB), and 5,5′-dithiobis-(2-nitro benzoic acid) (DTNB), were purchased from Sigma Aldrich, USA. ELISA kits for NF-kB (CAT # PRS-2064Mo), TNF-α (CAT# PRS-30651Ra), PPAR-γ (CAT# PRS-30503Ra) and COX-2 (CAT# PRS-30205Ra) were acquired from Nanjing Pars Biochem CO., Ltd China.

## 3. Materials and methods

### 3.1. Animals and ethical approval

The study employed adult male Sprague Dawley rats weighing 180-250g. They were habituated under lab conditions at temperature of 25–30°C and controlled humidity with a 12 hr alternating light and dark cycle. All the animals had free access to food and water ad libitum. The experiments were carried out according to ARRIVE guidelines and by the approval of Research and Ethics Committee of Riphah Institute of Pharmaceutical Sciences, Riphah International University (Reference no.: REC/RIPS/2019/14).

### 3.2. Chronic constriction injury model

Chronic constriction injury was performed according to the previously described protocol, with slight modifications [18]. Briefly, the animals were sedated using a cocktail of xylazine and ketamine (1:10 mg/kg body weight). We have applied all the laboratory procedures to minimize rat sufferings, such as heating pad, sterilization and fluid replenishment with normal saline. The left leg was shaved and disinfected using 70% ethanol and an incision was made 3-4mm below femur and sciatic nerve was exposed. Using four loose ligatures, the sciatic nerve was constricted at 1 mm distance and the muscle and skin were sutured again using 3.0 silk. The animals that shows sign of constriction injury were included in the experiment.

### 3.3. Study design and drug treatment

**Fig 1:**
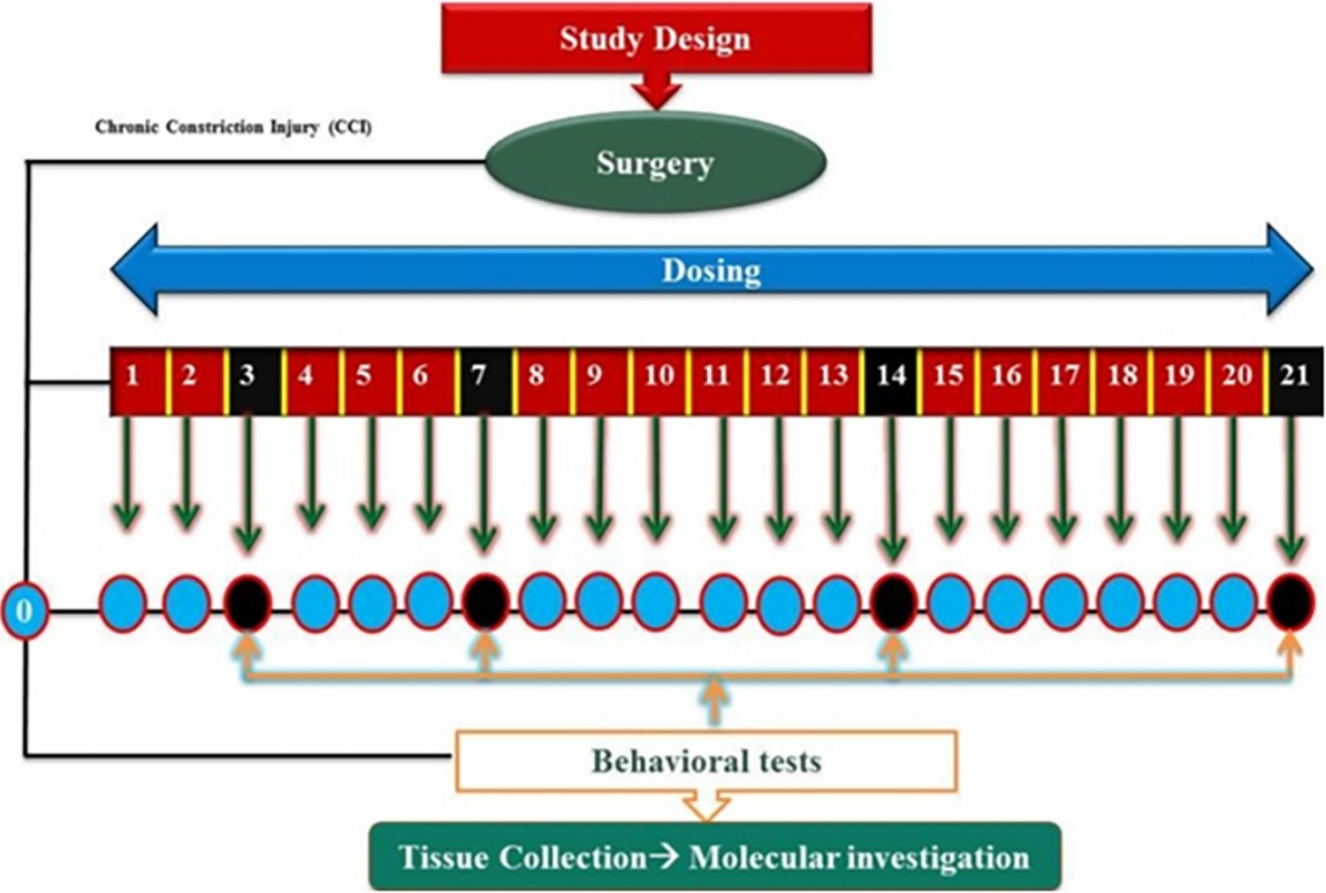
Scheme of the experimental work.

Animals were randomly divided into five groups with n = 6 in each group as follows: Group 1 (Sham group): the animals in this group underwent complete protocol of surgery except for sciatic nerve ligation; Group 2 (disease group; CCI + Saline): animals in this group underwent CCI surgery; Group 3 (CCI + Pioglitazone group): the animals in this group received pioglitazone intraperitoneally at a dose of 10 mg/kg after CCI surgery; Group 4 (CCI + Emodin-treatment group): the animals in this group received Emodin intraperitoneally at a dose of 50 mg/kg after CCI surgery; group 5 (CCI + Emodin + GW9662 group) the animals in this group received inhibitor GW9662 2 mg/kg, after 30 minutes of Emodin treatment. All the dosing was done 24 hrs after CCI surgery, daily for 21 days, at the same time. Behavioral evaluation was carried out at day 3,7,14 and 21 after CCI surgery **(Fig 1).**

### 3.4. Behavioral evaluation

#### 3.4.1 Mechanical allodynia

To assess mechanical deformity, mechanical allodynia was carried out using Von Frey hairs technique. Post acclimatization, animals were placed in an enclosed chamber having mesh flooring and Von Frey filaments of 1, 2, 4, 6, 8, 10, 15 and 20 g were gradually applied at low pressure, in such a way that the filament bent for 2–3 seconds, onto the planter surface of the animal’s feet. A quick retreat or paw-licking was noted to detect mechanical allodynia [19].

#### 3.4.2. Cold allodynia

To assess the sciatic nerve damage, cold allodynia was performed as previously described. Post acclimatization, animals were placed on a cold surface with 4°C temperature and thermal hyperalgesia was measured for 20 s each. Five reading were obtained with a 5 minutes interval between each [20].

#### 3.4.3. Walk track analysis

To assess the functional deformity following CCI, walk track analysis was carried out according to the previous protocols [18]. It was determined by dipping the hind paw of the rat in ink and then letting him walk on a white piece of paper. The footprints of all animals in a group were then evaluated for paw deformities.

### 3.5. Biochemical evaluation

After anesthetizing rats using a cocktail of xylazine (9 mg/kg) and ketamine (90 mg/kg) intraperitoneally, rats were decapitated following AVMA guidelines. For biochemical evaluation, the ipsilateral sciatic nerve and spinal cord were isolated on 21^st^ day after behavioral evaluation and homogenized in buffer (pH = 7.4) containing phenylmethylsulfonyl fluoride (PMSF) as a protease inhibitor [21]. The supernatant was collected and processed for further biochemical investigation.

### 3.6. Antioxidant assays

#### 3.6.1 Estimation of glutathione S-Transferase and Reduced glutathione (GST and GSH)

To detect the levels of oxidative stress caused by CCI, GST and GSH analysis was carried out using previously mentioned protocol [22, 23]. GST activity was determined by estimation of CDNB conjugate formation using 0.1 M phosphate Buffer and tissue homogenate and measuring the absorbance at 340 nm using Microplate reader (BioTek ELx808, Winooski, VT) for 5 minutes (23°C). GST activity was expressed as μmol of CDNB conjugate/min/mg of protein [22]. GSH activity was determined using previously established protocols [23] where GSH was oxidized with DTNB and 0.2 M Phosphate buffer with 0.2 ml of tissue supernatant for 10 mintes producing a yellow end product called 2-nitro-5-thiobenzoic acid. The absorbance of reaction mixture was measured at 412 nm. DTNB solution without tissue lysate and phosphate buffer were kept as blank and control, respectively. GSH actvity was expressed in units of μmol/mg of proteins.

#### 3.6.2. Estimation of catalase (CAT)

In the presence of catalase, the rate of H_2_O_2_ degradation was measured. With the use of a microplate reader, the absorbance at 240 nm was calculated. Catalase activity was measured in moles H_2_O_2_/min/mg of protein [23].

#### 3.6.3. Estimation of Lipid Peroxidation (LPO)

Lipid peroxidation following CCI was estimated using thiobarbituric acid reactive substances (TBARS) by calorimetric method [24, 25]. Briefly, tissue homogenate was treated with Ferric chloride, Ascorbic acid and phosphate buffer and incubated for 1 hour at 37°C. 10% trichloroacetic acid and 0.66% thiobarbituric acid were then added to the solution to halt the reaction and the TBARS concentration was measured at 535 nm in units of nmol/min/mg of proteins using ELISA Microplate reader.

#### 3.6.4. Estimation of nitric oxide (NO)

As per earlier protocols, NO assay was carried out. In distilled water, 50 mL of tissue supernatant was combined with an equal amount of saline and Griess reagent. The mixture was incubated for thirty minutes at room temperature. Using an ELISA plate reader and standard sodium nitrite solution as a reference, the absorbance of the resulting mixture was measured at 546 nm [26].

### 3.7. Hematoxylin and Eosin (H & E) staining

Five rats from each group were used to conduct the morphological examination. The spinal cord and sciatic nerve tissues were fixed in 10% paraformaldehyde until further study. Using a rotary microtome, the tissues were sliced into 5-mm thin slices, which were stained with hematoxylin and eosin (H&E). Under an optical microscope, pictures were obtained of the tissues, as described [27].

### 3.8. ELISA (Enzyme-Linked Immunosorbent Assay)

According to the manufacturer guidelines, the following markers were detected: tumor necrotic factor (TNF-α), cyclooxygenase-2 (COX-2), phosphorylated nuclear factor kappa B (p-NFkB) and peroxisome proliferator-activated receptors (PPAR-γ). The spinal cord and sciatic nerve tissues (n = 5/group) were homogenized at 15×1000 rpm using a SilentCrusher M (Heidolph) and centrifuged (at 1350 g for 1 hour) before the supernatant was collected. The supernatant was then tested using a Rat ELISA kit for the measurement of p-NFkB (CAT # PRS-2064Mo), TNF-α (CAT# PRS-30651Ra), PPAR-γ (CAT# PRS-30503Ra) and COX-2 (CAT# PRS-30205Ra)

### 3.9. RT-PCR analysis (Real Time Polymerase Chain Reaction)

Following homogenization of the spinal cord and sciatic nerve tissues (n = 5/group), total ribonucleic acid (RNA) was extracted using the trizol technique in accordance with the manufacturer’s instructions. Reverse transcriptase was used to generate cDNA from 1–2 μg of total RNA, and real-time PCR was used to amplify the cDNA using a thermocycler. The expression levels of beta-actin were used to normalize the mRNA expression. The 2^^ΔΔ-CT^ method for real-time quantitative PCR was used to calculate the relative gene expression [13]. Primers sequences for GAPDH and PPAR-γ are as follows:

GAPDH forward: CATCACTGCCACCCAGAAGACTG

GAPDH reverse: ATGCCAGTGAGCTTCCCGTTCAG

PPAR-γ forward: CCCTTTACCACGGTTGATTTCTC

PPAR-γ reverse: GCAGGCTCTACTTTGATCGCACT

### 3.10. Statistical analysis

All the data is expressed as mean ± SEM. For oxidative stress markers, One-Way ANOVA followed by *post-hoc* tukey’s test was applied using GraphPad prism 8. ELISA and behavioral analysis was carried out using Two-Way ANOVA with *post-hoc* Tukey’s test. Symbols # or * represent significant difference values P < 0.05, ## or ** represent P < 0.01, and ### or *** represents P < 0.001 values for significant differences.

## 4. Results

### 4.1. Effect of emodin on mechanical hyperalgesia in neuropathic rats

**Fig 2:**
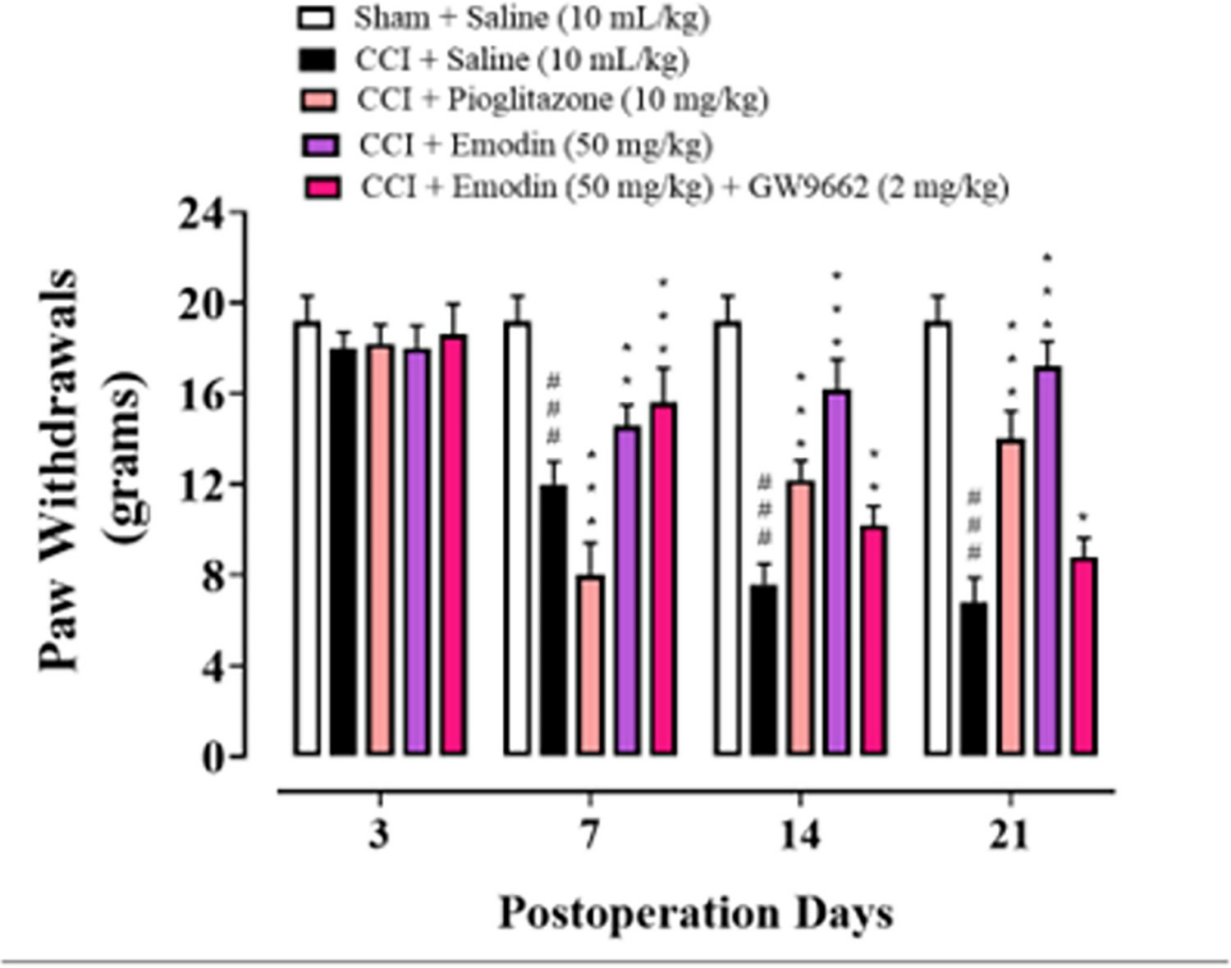
Graphical representation of the effect of emodin and pioglitazone on mechanical allodynia on 0, 7, 14 and 21^th^ day. Data expressed as mean ± SEM, (n = 6).Two-way ANOVA followed by post-hoc Tukey test. ^###^*P* < 0.001 vs. sham and *P < 0.05, **P< 0.01, ***P < 0.001 vs. chronic constriction injury (CCI).

Prior to surgery, all rats exhibited comparable reactivity to mechanical stimuli. CCI induced significant mechanical hyperalgesia in the hind paw of rats postoperatively, on 7th day, and the effects persisted for 21 days. As compared to saline-treated animals(10 mL/kg), administration of pioglitazone (10 mg/kg) and emodin (50 mg/kg) significantly delayed the onset of mechanical hyperalgesia in rats with CCI (*P* < 0.001). Furthermore, the PPAR-*γ* antagonist GW9662 (2 mg/kg, i.p.) along with emodin counteracted the analgesic effects of emodin **(Fig 2).**

### 4.2. Effect of emodin on cold hyperalgesia of neuropathic rats

**Fig 3:**
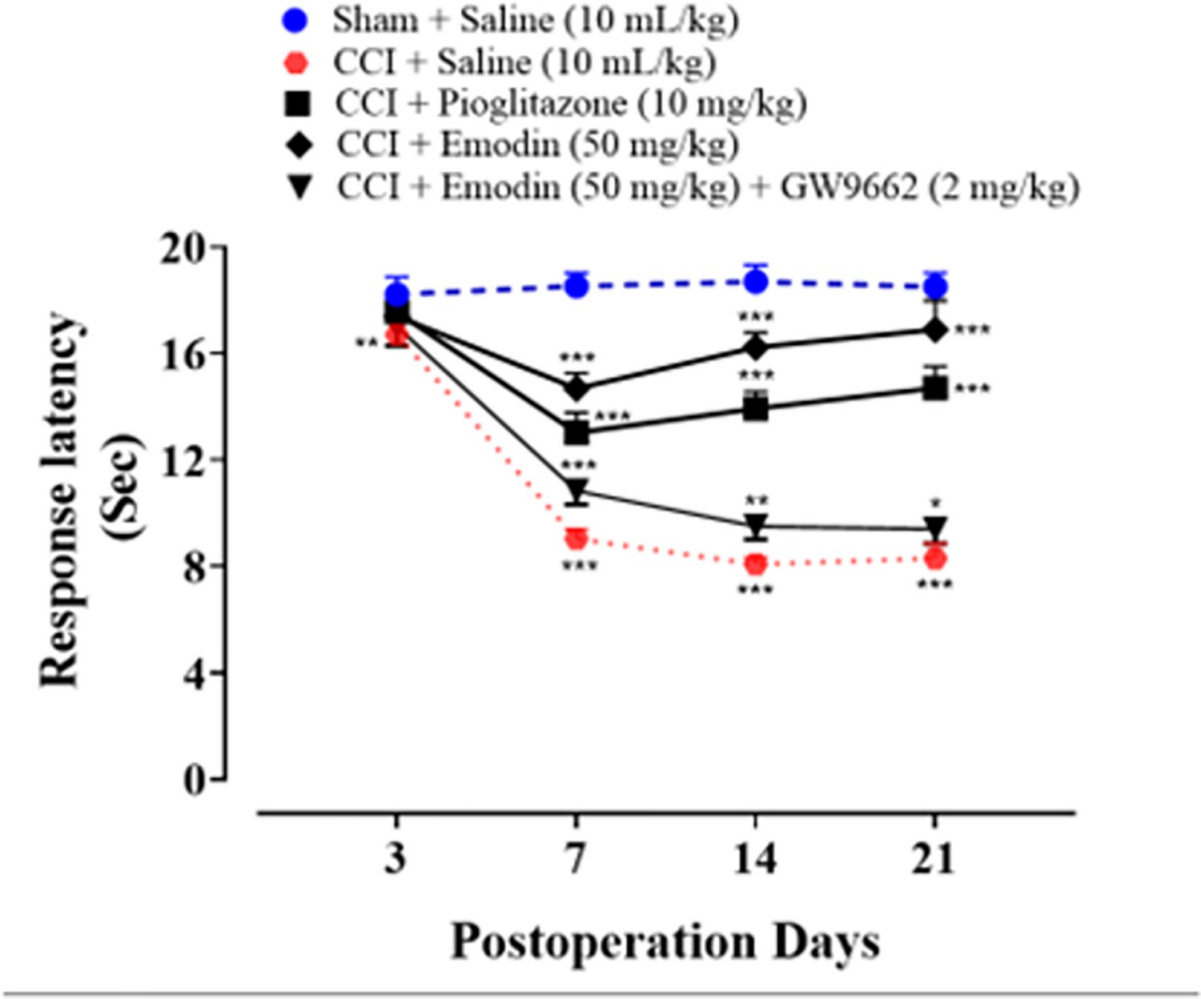
Graphical representation of the effect of emodin and pioglitazone on cold allodynia on 3, 7, 14 and 21^th^ day. Data expressed as mean ± SEM, (n = 6). Two-way ANOVA followed by post-hoc Tukey test. ^###^*P* < 0.001 vs. sham and *P < 0.05, **P < 0.01, ***P < 0.001 vs. chronic constriction injury (CCI).

On postoperative day 7, it was observed that CCI caused significant cold allodynia in the ipsilateral hind paw of rats, which persisted till postoperative day 21. In comparison to rats treated with Saline (10 mL/kg), both pioglitazone (10 mg/kg) and emodin (50 mg/kg) elevated response latency on days 7, 14, and 21 significantly (*P* < 0.001), while co-treatment of GW9662 (2 mg/kg, i.p.) with emodin resulted in reduced analgesic efficacy of emodin (**Fig 3**).

### 4.3. Effect of emodin on walk-track analysis

**Fig 4:**
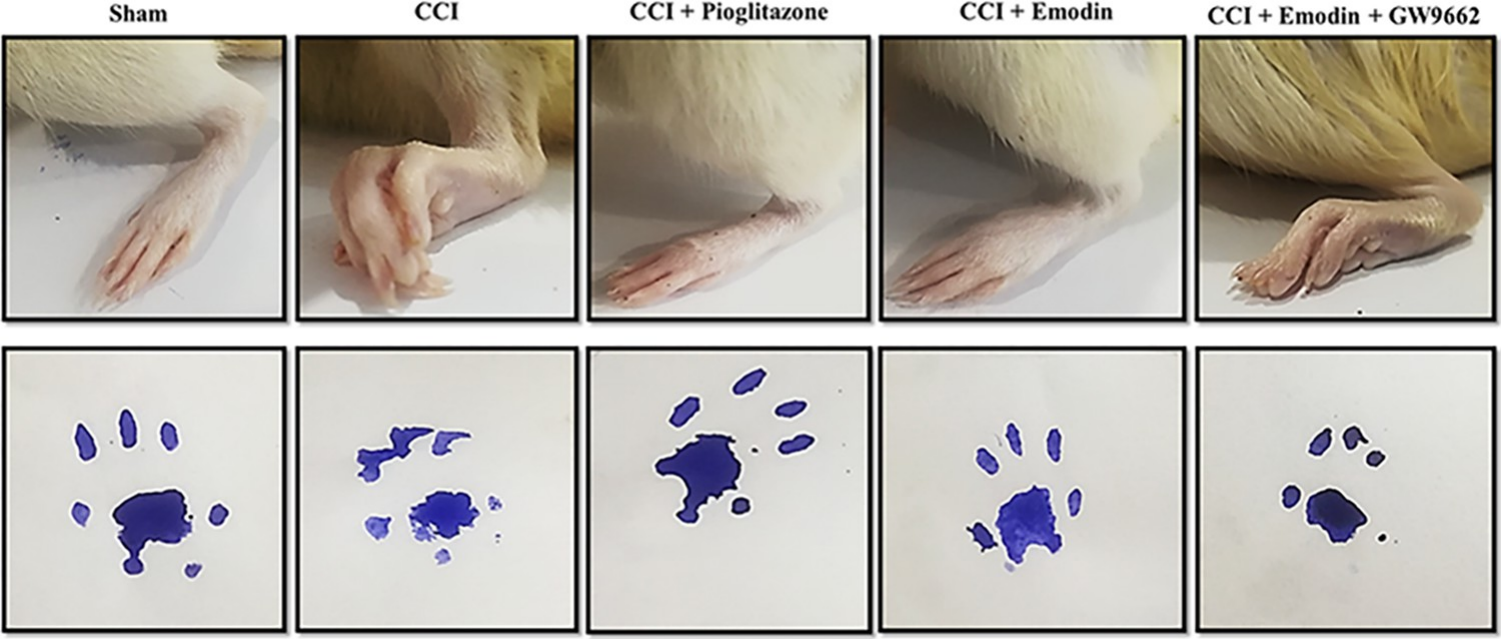
Represents the effect of emodin and pioglitazone on paw deformation in chronic constriction injury.

Walk-track analysis was performed to study the effect of emodin in contrictive injury. The sham group showed normal gait and regular pawprints. After constrictive injury, the animals were unable to rest their paws on a smooth surface normally since it was significantly afflicted by the condition. **Fig 4** illustrates how treatment with pioglitazone (10 mg/kg) and emodin (50 mg/kg) reduced the CCI-induced paw deformation and gait abnormality. CCI-induced paw deformity was not ameliorated by co-treatment of emodin with GW9662 (2 mg/kg, i.p.) **(Fig 4).**

### 4.4. Effect of emodin on antioxidant profile of neuropathic rats

**Fig 5:**
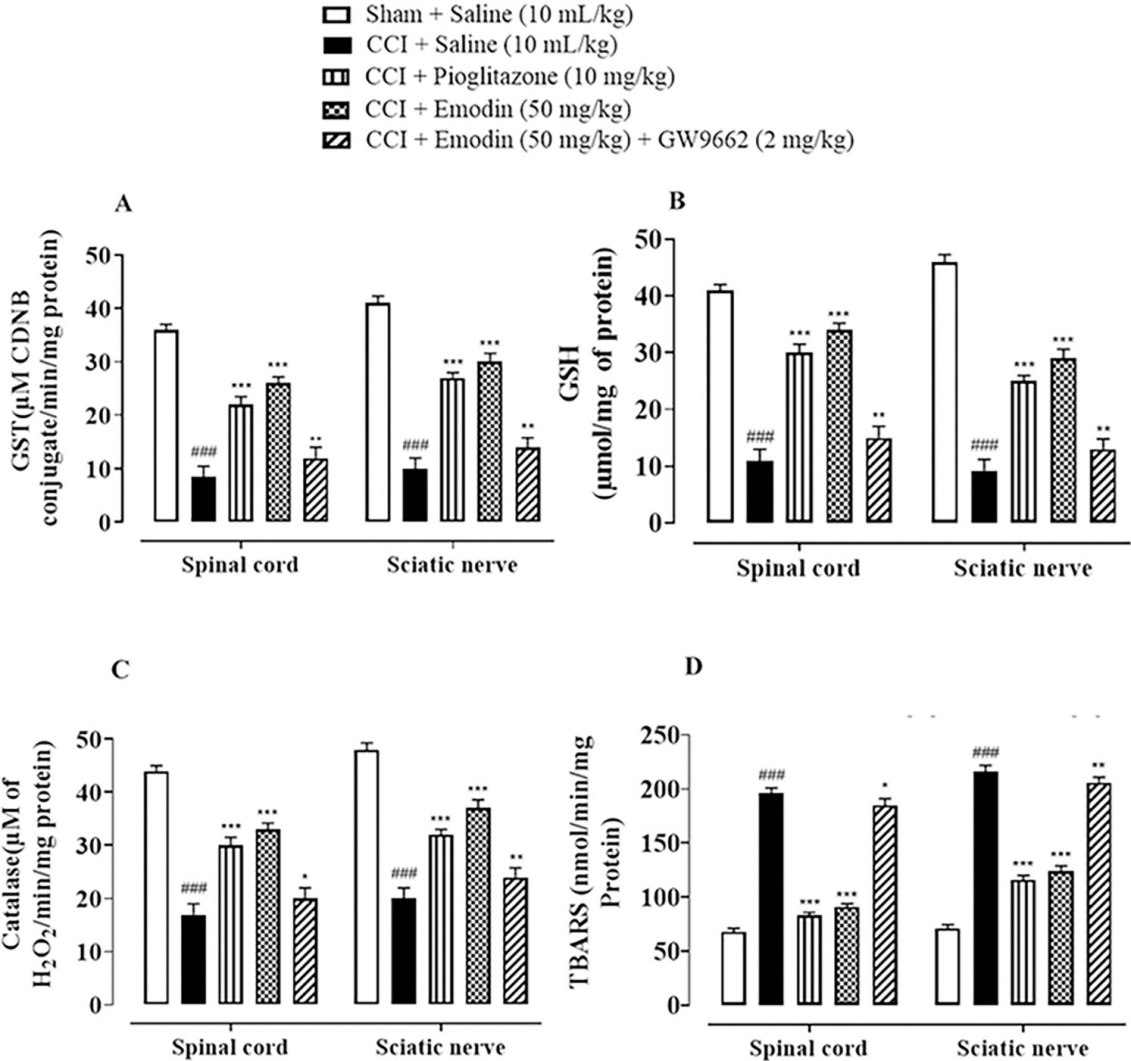
Graphical representation of the effect of Emodin and Pioglitazone against **(A)** glutathione-S-transferase (GST), **(B)** reduced glutathione (GSH), **(C)** catalase and **(D)** lipid peroxidation (LPO) in CCI treated rats’ spinal cord and sciatic nerve tissues. Values expressed as mean ± SEM (n = 5/group). One-way ANOVA with *post-hoc* Tukey’s test. ^###^*P* < 0.001 vs. sham and *P< 0.05, **P < 0.01, ***P< 0.001 vs. chronic constriction injury (CCI).

Antioxidant profile of the rats was evaluated by analysing GST, GSH, and catalase activity post treatment. The activities of GST, GSH and catalase were significantly replenished in spinal cord and sciatic nerve tissues of the disease group, while an increased lipid peroxidation was observed, depicting oxidative damage. Both pioglitazone and emodin treatment significantly reduced lipid peroxidtion while restoring the antioxidant’s levels. Whereas, GW9662 (2 mg/kg, i.p.) barely altered the oxidative profile of the emodin-treated animals (**Fig 5**).

### 4.5. Effect of emodin on NO levels in neuropathic rats

NO-levels were significantly elevated in CCI + Saline treated tissues. Treatment with emodin and pioglitazone significantly restored the levels of NO in sciatic nerve and spinal cord tissues as shown in **Table 1A** and **1B**). Co-treatment of GW9662 and emodin produced minimal alterations.

**Table 1:** A and B represents the effect of emodin on expression of nitric oxide (NO) in spinal cord and sciatic nerve.

A Spinal cord	
**Groups**	**NO (μmol/mg of Protein)**
Sham + Saline (10 mL/kg)	41.45 ± 2.1
Chronic constriction injury (CCI) + Saline (10 mL/kg)	132.23 ± 2.3^###^
CCI + Pioglitazone (10 mg/kg)	63.32 ± 1.5***
CCI + Emodin (50 mg/kg)	75.55 ± 1.8***
CCI + Emodin (50 mg/kg) + GW9662 (2mg/kg)	109.12 ± 2**
**B Sciatic nerve**	
**Groups**	**NO (μmol/mg of Protein)**
Sham + Saline (10 mL/kg)	38.35 ± 1.1
Chronic constriction injury (CCI) + Saline (10 mL/kg)	125.43 ± 2.4^###^
CCI + Pioglitazone (10 mg/kg)	71.56 ± 1.3***
CCI + Emodin (50 mg/kg)	82.11 ± 1***
CCI + Emodin (50 mg/kg) + GW9662 (2mg/kg)	115.12 ± 2*

The data were expressed as the mean ± SEM, (n = 6). One way ANOVA followed by *post-hoc* Tukey test. ^###^*P* < 0.001 vs. sham

*P < 0.05

**P < 0.01 and

***P < 0.001 vs. chronic constriction injury (CCI).

### 4.6. Effects of emodin on histopathological damages in neuropathic rats

**Fig 6:**
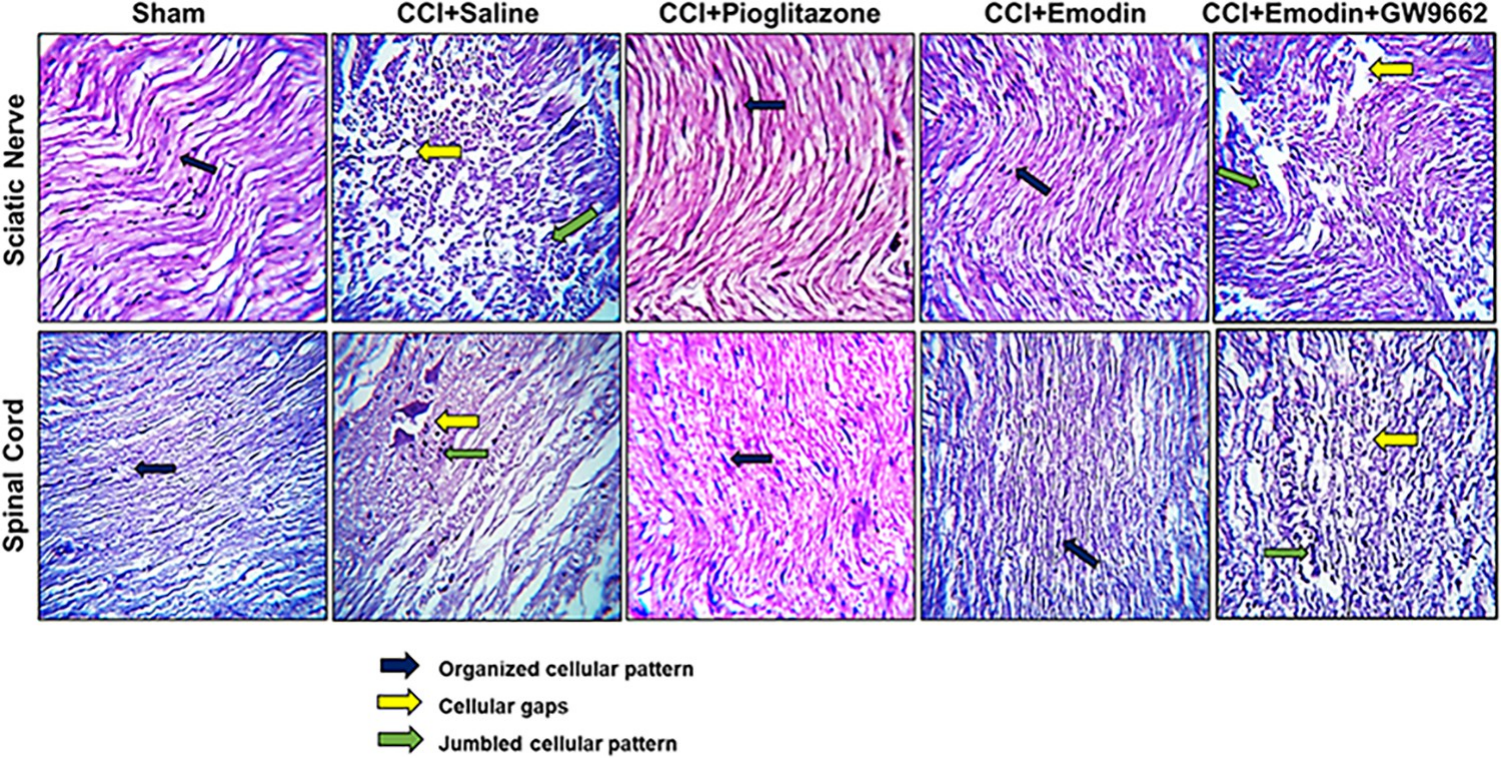
Histopathological slides showing effect of emodin and pioglitazone in CCI treated rats spinal cord and sciatic nerve tissues using hematoxylin and eosin staining histopathological technique. Bar 50 μm, magnification 40 x.

CCI inflicted significant pathological alteration in animals which were observed in both sciatic nerve and spinal cord. Different signs of nerve injury were observed as cellular spaces, edema development, and disorganized cellular architecture. Treatment with Emodin and pioglitazone significantly improved the disorientation of the sciatic nerve and spinal cord, while co-treatment with GW9662 reduced the repairing ability of emodin (**Fig 6**).

### 4.7. Effect of emodin on inflammatory markers

**Fig 7:**
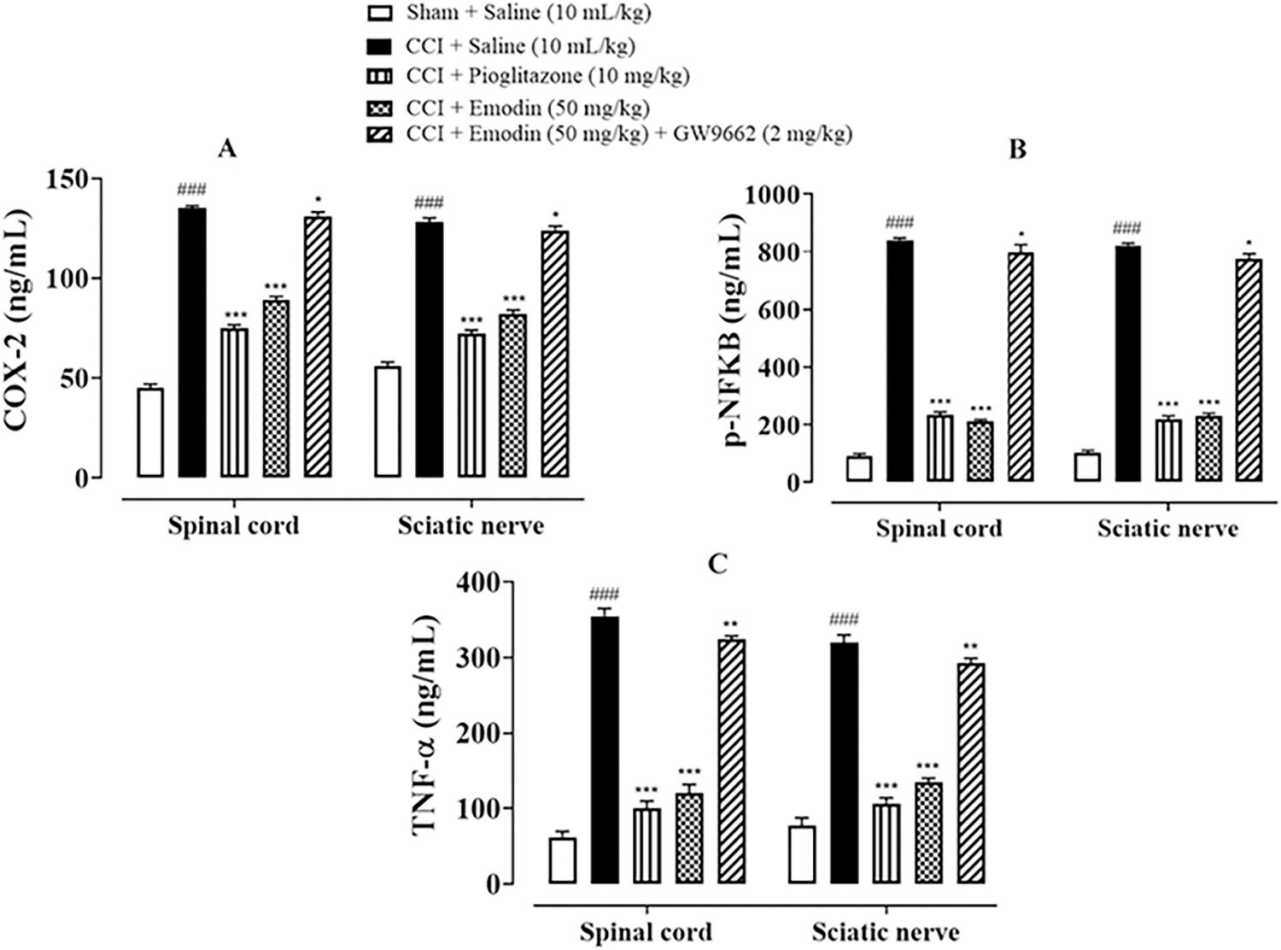
Effect of Emodin and Pioglitazone against (**A**) cyclooxygenase-2 (COX-2), (**B**) phosphorylated-nuclear factor kappa B (p-NFƙB) and (**C**) tumor necrosis factor alpha (TNF-α) in CCI treated rats’ spinal cord and sciatic nerve tissues measured by enzyme-linked immunosorbent assay (ELISA) technique. Values expressed as mean ± SEM (n = 5/group). Two-way ANOVA with *post-hoc* Tukey’s test. ^###^*P* < 0.001 vs. sham and *P< 0.05, **P < 0.01, ***P< 0.001 vs. chronic constriction injury (CCI).

As shown in **Fig 7A–7C**, the effects of Emodin on the expression of various inflammatory markers as COX-2, TNF-α and p-NFkB in sciatic nerve and spinal cord were studied. Results indicated hyper-expression of inflammatory markers (COX-2, TNF-α and p-NFκB) owing to induced inflammation by CCI (^###^*P* < 0.001 vs. sham). Treatment with Pioglitazone (10 mg/kg) and Emodin (50 mg/kg) significantly ameliorated the inflammatory status of the sciatic nerve and spinal cord (***P< 0.001 vs. CCI) while co-treatment with GW9662 reverted the ameliorative ability of emodin in neuronal inflammation (*P< 0.05, **P < 0.01 vs. CCI).

### 4.8. Role of PPAR-γ in the neuroprotective potential of emodin

**Fig 8:**
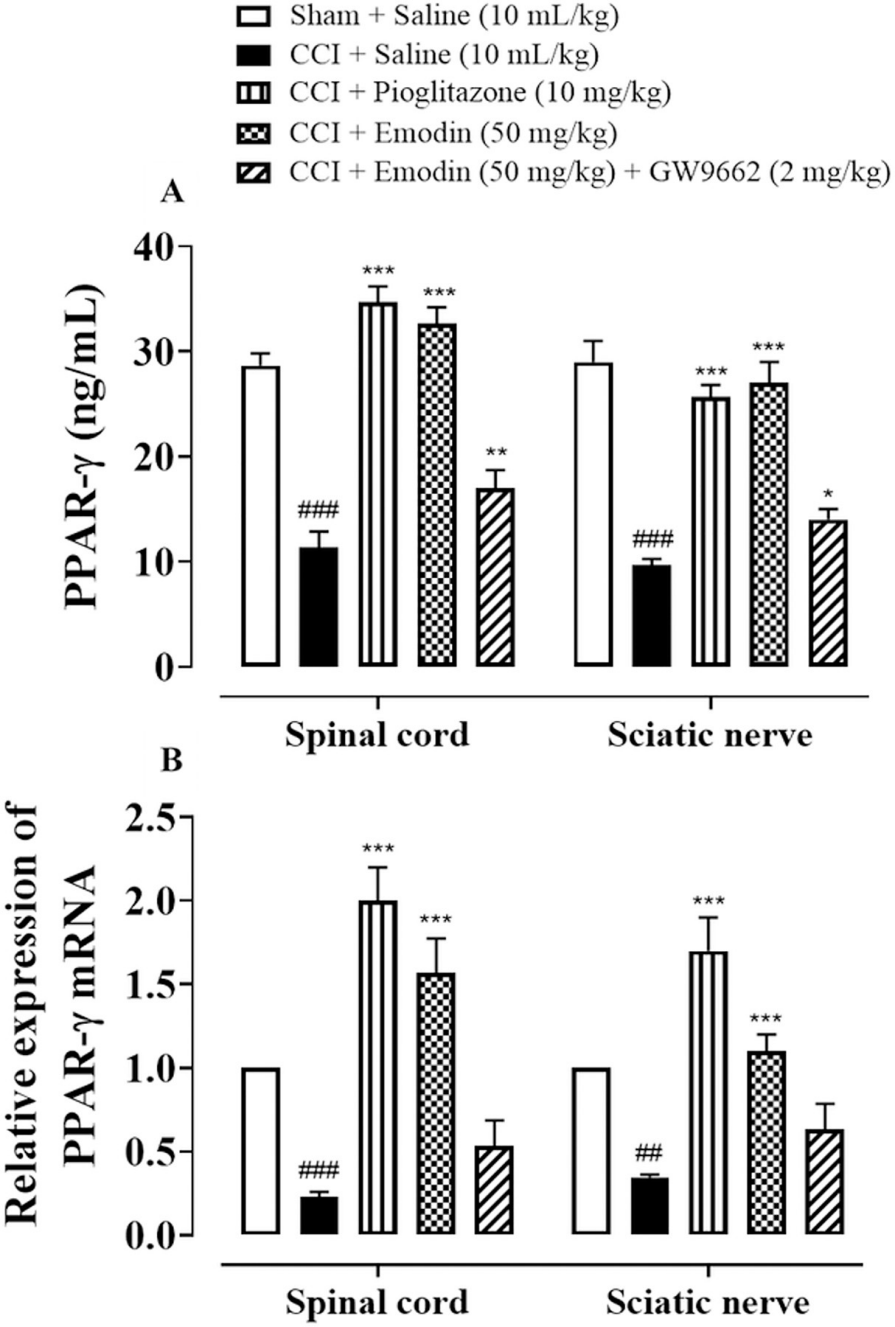
Effect of Emodin and Pioglitazone against (**A**) Peroxisome proliferator-activated receptors (PPAR-γ), by ELISA (**B**) RT-PCR. Values expressed as mean ± SEM (n = 5/group). Two-way ANOVA with *post-hoc* Tukey’s test. ^###^*P* < 0.001 vs. sham and *P< 0.05, **P < 0.01, ***P < 0.001 vs. chronic constriction injury (CCI).

The involvement of PPAR-γ in the neuroprotective ability of emodin was quantified through ELISA and RT-PCR (**Fig 8**). After CCI, animals demonstrated significant reduction in the levels of PPAR-γ as observed by both ELISA and RT-PCR. Pioglitazone (10 mg/kg) and Emodin (50 mg/kg) treatment significantly up-regulated PPAR-γ levels as compared to disease treated group (*P* < 0.001), while co-administration of GW9662 with emodin mitigated the restorative potential of emodin treatment.

## 5. Discussion

NP has been characterized by a chronic nervous system disorder, mainly presented with clinical manifestations of hyperalgesia or allodynia along with other atypical paresthesia in response to pernicious stimuli. With a high prevalence rate [28] and the heterogenicity of disease, the underlying mechanisms still remain unclear and NP still remains refractory to the present therapeutic regime. The current study determined that the pretreatment of emodin alleviates the behavioral alterations associated with pain sensitivity; and suppress the inflammatory mediators TNF-α, COX-2 and NF-kB triggered by NP caused by chronic constrictive injury. Emodin reduced NP-induced LPO levels in addition to regulating NO levels. It also elevated the body’s natural oxidative response by activating GST, GSH, and CAT. Through RT-PCR analysis, ELISA and a PPAR- γ blocker, the present study additionally investigated the involvement of the PPAR- γ pathway in the therapeutic impact of emodin on NP produced by CCI. The results of the present investigation may aid in not only clarifying how PPAR-γ is involved in NP treated by emodin, as well as providing a new therapeutic option for the treatment and management of NP.

Utilization of plant-derived natural compounds for the treatment of various diseases is on a rise, owing to their low toxicity profile, minimal side effects and enhanced therapeutic potential. Emodin, an anthraquinone, is an active Rhubarb constituent and has been extensively studied as anti-cancer, anti-mutagenic, anti-ulcer, anti-apoptotic, anti-inflammatory and immunosuppressant [12–15]. Various studies have also reported the ameliorative effect of emodin on peripheral NP [29]. However, none of the study suggest the involvement of PPAR-γ pathway for the alleviation of NP by emodin. The current study outlines the involvement of PPAR-γ pathway in the mediation of pain transmission caused by emodin in CCI-induced NP.

In this study, a CCI model was established that serves to induce chronic NP by ligation of sciatic nerve causing peripheral nerve injury. This results in pain hypersensitivity and sensorimotor dysfunctions along with behavioral alterations as social interaction, sleep abnormalities and anxiety and depression-like symptoms [30, 31]. In consistence with previous studies, our results demonstrated a robust pain hypersensitivity following CCI, measured by mechanical allodynia and cold allodynia along with paw deformity [32]. The present study however, indicated that emodin treatment delayed the animal’s responsiveness to the mechanical stimulation measured by paw withdrawal, with Von-Frey filaments, and caused a significantly delayed response latency in cold allodynia. Also, as a result of emodin treatment, paw deformity of the animals improved significantly. As previously demonstrated, Pioglitazone administration enhanced the animals’ pain hypersensitivity which was mitigated by GW9662 and emodin co-treatment.

Oxidative stress has been studied extensively as an underlying pathophysiology in most of the neurological diseases. Nerve ligation in CCI-model causes generation of free radicals which further damage nerve tissues resulting in progression and worsening of disease along with hyperalgesia and other pain-associated consequences [18, 33]. Emodin treatment attenuated the enhanced LPO and NO levels and upregulated the diminished GST, GSH and CAT levels which is also in line with previous studies [19, 34]. Hence, we can deduce that antioxidant effect of emodin also played a vital role in ameliorating pain and its related abnormalities by preventing oxidative damage to the nerve.

There is increasing evidence of the role of inflammatory process in NP. Under physiological conditions, inflammation is a process by which body responds to injurious stimulus and initiate repair. Disturbances in the neuroinflammatory mechanisms may lead to chronic pain [35]. Nonetheless, recent studies have clarified the role of immune system components and glial cells besides neuronal pathways, which not only trigger neuroinflammation but also progress NP [36]. It involves both immune cell activation and mediator release. As NP may arise as a result of lesions as spinal injury or traumatic neuropathy, hence, excessive inflammation may be a hallmark of NP which results in generation and maintenance of persistent pain [3]. Peripheral nerve damage leads to local inflammatory response causing activation of microglia, astrocytes and Schwan cells causing release of pro-inflammatory cytokines as IL-6, TNF-α and IL-1β which further activates inflammatory cytokines as COX-2 and NF-kB [37]. Their activation is also associated with indirect nociception by further promoting synthesis of inflammatory mediators [38] and direct nociception by sensitizing nociceptors [39]. Our results were also in accordance with previous researches and showed an enhanced inflammation after CCI-induced NP which was ameliorated after Emodin and Pioglitazone treatment and blockade of PPAR- γ pathway by GW9662 significantly mitigated the anti-inflammatory potential of emodin.

Growing evidence points to the role of PPAR- γ in the reduction of NP by enhancing pain hypersensitivity after traumatic nerve injury and suppressing astrocyte activation. (10). Furthermore, the possibility of using pioglitazone-activated PPAR-γ as a therapeutic drug to treat orofacial NP has been investigated [40]. Numerous recent research reveal that activating PPAR- γ has been linked to the inhibition of neuroinflammatory mediators like NF-kB, which prevents the activation of microglia and further prevents the production of TNF-α and IL-1 [40, 41]. In models of sciatic nerve damage, PPAR- γ activation has also been linked to decreased mechanical allodynia and thermal hyperalgesia [42]. Similarly, our findings showed better NP and related processes after pioglitazone administration with Emodin therapy, demonstrating a significant NP mitigation. Moreover, the use of the PPAR- γ inhibitor GW9662 proved that the PPAR-γ pathway was involved for Emodin’s therapeutic effects on NP and neuroinflammation.

## 6. Conclusion

**Fig 9:**
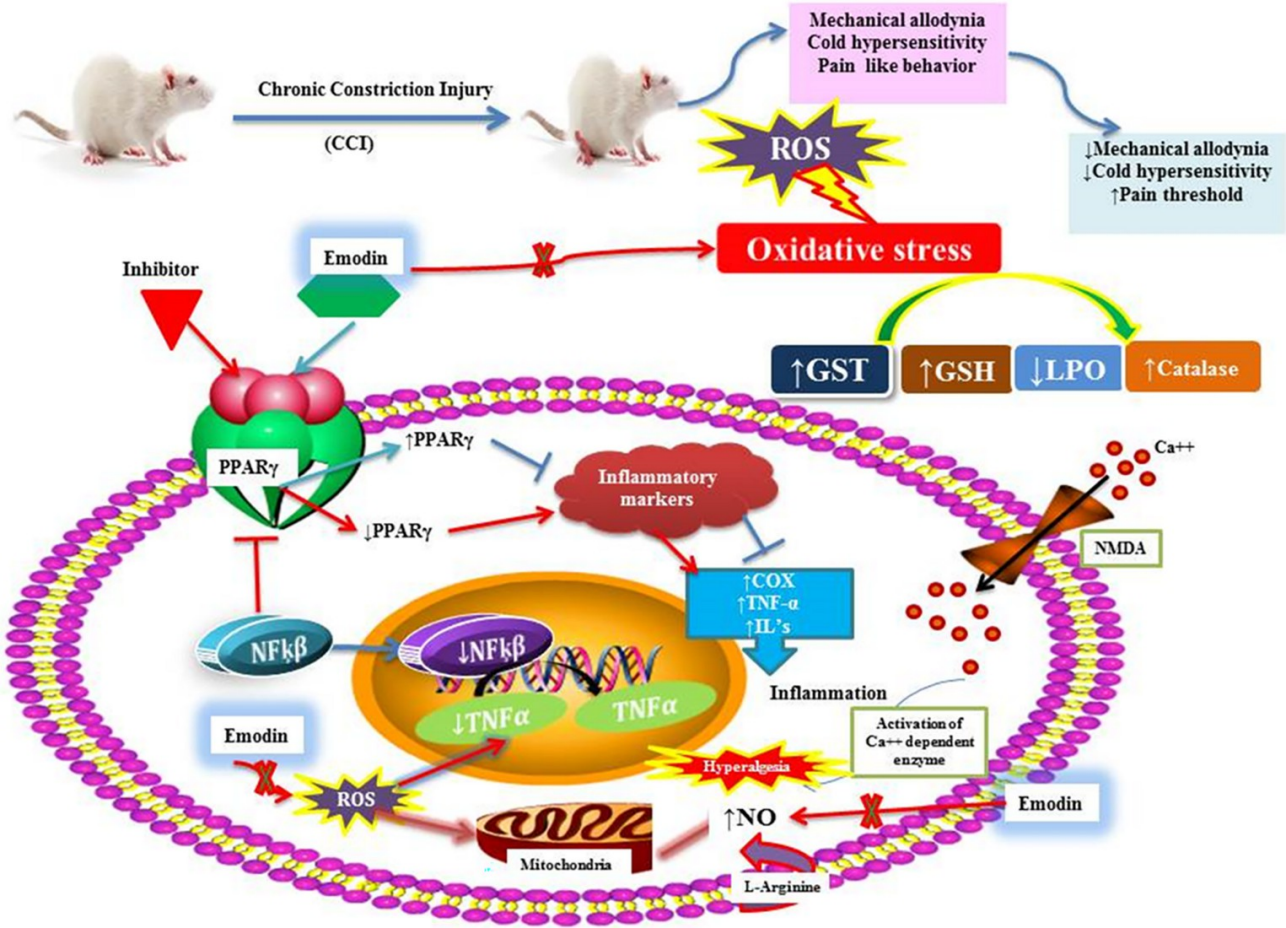
Possible mechanism underlying the antioxidant, analgesic and anti-inflammatory potential of emodin in CCI-induced NP.

In conclusion, our study demonstrated that emodin treatment projected an ameliorative effect towards pro-inflammatory and inflammatory cytokines as NF-kB, TNF-α and COX-2 and relatively suppressed pain hypersensitivity and improved the oxidative stress levels following CCI-induced NP. Furthermore, we suggested the involvement of PPAR-γ pathway in the therapeutic potential of emodin in chronic nerve injury, thereby presenting emodin as a probable therapeutic candidate to mitigate NP via modulating PPAR-γ pathway. However, since the mechanism of NP is heterogenous, further research is needed to elucidate the likely pathways by which emodin tends to exert its therapeutic impact in NP and related neuroinflammation, as the results of this study merely provide a possible mechanism (**Fig 9**).

## 7. Institutional review board statement

The experiments were carried out according to ARRIVE guidelines and by the approval of Research and Ethics Committee of Riphah Institute of Pharmaceutical Sciences, Riphah International University (Reference no: REC/RIPS/2019/14).
