# Expression of Concern: Emodin alleviates chronic constriction injury-induced neuropathic pain and inflammation via modulating PPAR-gamma pathway

**DOI:** 10.1371/journal.pone.0328450

**Published:** 2025-07-17

**Authors:** The PLOS One Editors

After this article [1] was published, the following concerns were noted:

- The article cited as Reference 21 was retracted after [1] was published.
- The article does not comply with the PLOS Data Availability policy.
- Many of the references are incomplete and do not list necessary information such as journal names.
- PLOS has concerns about aspects of the article’s [1] authorship and peer review.

Reference 21 is replaced with [2].

The article’s Data Availability statement was incorrect and is updated to: All relevant data are available from the authors upon request.

In light of the cumulative issues, the *PLOS One* Editors issue this Expression of Concern.
